# Fer governs mTORC1 regulating pathways and sustains viability of pancreatic ductal adenocarcinoma cells

**DOI:** 10.3389/fonc.2024.1427029

**Published:** 2024-08-14

**Authors:** Ilan Schrier, Orel Slotki-Itzchakov, Yoav Elkis, Nofar Most-Menachem, Orit Adato, Debora Fitoussi-Allouche, Sally Shpungin, Ron Unger, Uri Nir

**Affiliations:** ^1^ Department of Surgery, Rabin Medical Center, Petah Tikva, Israel; ^2^ Sackler Faculty of Medicine, Tel Aviv University, Tel Aviv, Israel; ^3^ The Mina and Everard Goodman Faculty of Life-Sciences, Bar-Ilan University, Ramat-Gan, Israel

**Keywords:** Fer, E260, pancreatic ductal adenocarcinoma, mitochondria, AMPK, mTORC1

## Abstract

Pancreatic ductal adenocarcinoma (PDAC) is one of the deadliest cancers with a high percentage of morbidity. The deciphering and identification of novel targets and tools for intervening with its adverse progression are therefore of immense importance. To address this goal we adopted a specific inhibitor of the intracellular tyrosine kinase Fer, whose expression level is upregulated in PDAC tumors, and is associated with poor prognosis of patients. Subjecting PDAC cells to the E260-Fer inhibitor, unraveled its simultaneous effects on the mitochondria, and on a non-mitochondrial ERK1/2 regulatory cascade. E260 caused severe mitochondrial deformation, resulting in cellular- aspartate and ATP depletion, and followed by the activation of the metabolic sensor AMPK. This led to the phosphorylation and deactivation of the bona fide AMPK substrate, RAPTOR, which serves as a positive regulator of the mTORC1 metabolic hub. Accordingly, this resulted in the inhibition of the mTORC1 activity. In parallel, E260 downregulated the activation state of the ERK1/2 kinases, and their ability to neutralize the mTORC1 suppressor TSC2, thereby accentuating the inhibition of mTORC1. Importantly, both activation of AMPK and downregulation of ERK1/2 and mTORC1 were also achieved upon the knockdown of Fer, corroborating the regulatory role of Fer in these processes. Concomitantly, in PDAC tumors and not in healthy pancreatic tissues, the expression levels of Fer demonstrate moderate but statistically significant positive correlation with the expression levels of mTOR and its downstream effector LARP1. Finally, targeting the Fer driven activation of mTORC1, culminated in necrotic death of the treated PDAC cells, envisaging a new intervention tool for the challenging PDAC disease.

## 1 Introduction

Pancreatic ductal adenocarcinoma (PDAC) is the seventh deadliest cancer worldwide and the fourth deadliest in the developed world (1, 2), which often shows poor prognosis. Furthermore, curative treatment can rarely be applied because PDAC is often diagnosed at an advanced stage, and even after initial curative surgery the cancer often recurs. Thus, pancreatic cancer, for which the average overall 5-year survival is around 5%, is one of the most lethal malignancies (2).

Based on the described above, the need for innovative treatment remains urgent, and the quest for novel markers and targets for this challenging disease is preeminent. One of the proto-oncogenes whose high expression has been linked and associated with carcinogenesis, disease progression, metastatic dissemination, and poor prognosis of PDAC patients, is the intracellular tyrosine kinase - Fer (3). At the molecular level, Fer was shown to support the progression of PDAC through its engagement with the YY1/Fer/Stat3/MMP2 axis (4).

Fer is expressed in numerous somatic cells (5), and resides in various subcellular compartments. However, only in spermatogenic and cancer cells and not in normal somatic cells, Fer associates with complex 1 (Comp. I) of the mitochondrial electron transport chain (ETC) (6, 7). Thus, Fer is a cancer specific regulatory component of the reprogrammed mitochondria and metabolic system of malignant cells. Cancer cells exhibit upregulated aerobic glycolysis (the Warburg effect), an observation that led scientists to assume that mitochondrial oxidative phosphorylation (Oxphos.) is downregulated in all cancers. However, recent studies have shown that Oxphos. is also upregulated in certain cancers including PDAC tumors. Furthermore, evidence were provided that inhibition of mitochondrial Oxphos. efficaciously targets the viability of the challenging PDAC cells (8). Hence, the regulatory involvement of Fer in the reprogrammed mitochondrial metabolism and energy generation systems of malignant cells, portrays it as a potential targetable metabolic vulnerability factor that can be exploited for efficaciously and selectively intervene with the development and progression of PDAC tumors. Fer has also been shown to regulate breast cancer cell adhesion, migration and resistance to anoikis, and to be necessary for breast tumor growth and metastases formation in mice (9, 10). Similarly, Fer was shown to regulate the growth, migration and metastatic spreading of melanoma (11), non-small cell lung cancer (NSCLC), and ovarian cancer cells (12, 13). In addition to the case of PDAC (3), high expression of Fer was also found to serve as an independent prognostic factor that correlates with worse overall survival of triple negative breast cancer (TNBC) and NSCLC patients (7, 12, 13). It was also reported that a high expression level of Fer in clear renal cell carcinoma, is indicative of bad prognosis (14). Therein, correlation was documented between Fer expression and the size of the tumor, stage of the disease and metastases appearance. In liver cancer, Fer was found to play a key role in metastatic development and invasion, most probably through the stabilization and elevation of β-catenin phosphorylation (15). In compliance with these findings, Fer was also found to be abundant in prostate malignancies, in comparison to benign growths, and the knockdown of its level in these malignant cells was found to arrest cell division, and to hinder their ability to form colonies in soft agar (16). A high incidence of Fer mutations was found in colon cancer cells (17), and mesothelioma cells were found to express abnormal Fer activity (18). Collectively, Fer appears to be a malignancy promoting tyrosine kinase that is engaged in various regulatory processes in cancer cells.

The main aim of the current work was to decipher and identify tumor-promoting regulatory cascades and pathways in which Fer participates in PDAC cells, and to evaluate the therapeutic potential of a selective Fer inhibitor. As a major research tool we adopted the specific synthetic inhibitor of Fer-E260 that was developed in our lab and was found to be safe and non-cytotoxic toward primary human fibroblasts, epithelial cells, and hematopoietic stem cells (6). This approach unraveled mitochondrial and non-mitochondrial regulatory cascades that are regulated by Fer, and converge to a sustained activation of the key metabolic hub- mTORC1 (19, 20). mTORC1 was shown to direct numerous basic anabolic trajectories that propel malignant tumors development and progression (21, 22). Accordingly, targeting the Fer sustained activation of mTORC1, evoked the onset of necrotic death in E260 treated PDAC cells.

## 2 Materials and methods

### 2.1 Propagation of cell cultures

PANC-1 (CRL-1469) and SU.86.86 (CRL-18370) PDAC cells (human pancreatic ductal adenocarcinoma cells), were purchased from the American Type Culture Collection. The PANC-1 and SU.86.86 cells were cultured in DMEM (Sartorius 01–052-1A), and RPMI (Sartorius 01–100-1A), respectively, containing 10% FBS (Merck F0926) and 5% Penicillin-Streptomycin (Sigma-Aldrich P0781). Cells were grown at 37°C and in 5% CO_2_ in 10 cm plates. Cells where stripped using a 0.25% trypsin (Roche T4424) solution and re-introduced to fresh cultures once every 2–3 days.

### 2.2 Determination of cell viability

Determination of cell viability and death was conducted using the MultiTox-Fluor Multiplex Cytotoxicity Assay kit (Promega-G9200), in accordance with the manufacturer’s instructions. Cells were seeded in 96 well plates containing MEM growth medium (Sartorius 01–042-1A), supplied with 2 mM L-glutamine (Merck G7513), and E260 was added to them after 24 hr. Controls were treated with the E260 vehicle - 10% Cremophor (Merck 238470) + 10% ethanol, or 0.5% Polysorbate 80 (Tween -80, Sigma-Aldrich P4780). Plates were incubated for 24/48 hr, after which kit reagents were added for 1 hour in accordance with the manufacturer’s protocol. Florescence detects live cells at 400Ex/505Em wave-lengths and dead cells at 485Ex/520Em wave-lengths. The kit examines two protease activities simultaneously; one indicates live cells and the second indicates dead cells. Alternatively, viable and dead cells in a given culture, were counted using an automatic cell counter (Countess II, Life Technologies, Carlsbad, CA, USA), after the addition of Trypan blue to the sample.

### 2.3 Cell death analysis using annexin/PI staining

Cells (5×10^5^) were seeded in 6 cm cell culture dishes in MEM medium (Sartorius 01–042-1A) supplemented with 2mM L-glutamine (Merck G7513) for 24 hr. Cells were then treated with E260 at the indicated concentrations for 24 hr. Cells were stained with Annexin V-FITC and propidium iodide (PI) using the Annexin V-FITC Apoptosis Detection Kit (Abcam ab14085) following manufacturer’s instructions. Staining was quantified by FACS ARIAIII. All data were analyzed using FlowJo software (https://www.flowjo.com/solutions/flowjo).

### 2.4 Preparation of cell lysates

Cell lysates were prepared basically as described before (6). The cell medium was removed and the cells were rinsed once with PBS (Sartorius 02–023-1A). 750 μL of PBS was then added to the adherent cells on culture plates, and cells were stripped using a rubber policeman, and transferred to iced test tubes. This process was repeated twice, so in sum, cells were stripped using 1.5ml PBS. Cellular suspensions were centrifuged for 5 min at 500g at 4°C. Pellets were suspended in RIPA lysis buffer containing: 2mM Na_3_VO_4_ (Sigma-Aldrich 567540), 0.5% Doc (Sigma-Aldrich) 264103), 1% NP-40 (Merck), 20 mM TrisHCl (Sigma-Aldrich 93363) pH 7.5,150 mM NaCl pH 7.5 with added protease inhibitors (10 mg/ml Aprotinin, 20 mg/ml Benzamidine, 20 mg/ml Pefabloc and10 mg/ml Leupeptin, Merck PIC0002). Mixtures were incubated for 30 minutes, agitated at 4°C, and then centrifuged in an Eppendorf centrifuge for 10 minutes at 14000 rpm, at 4°C. The upper fluid containing proteins was taken for protein concentration determination, using the Bradford reagent (Bio-Rad-5000001.

### 2.5 Western-Blot analysis

Western-Blot (WB) analysis was performed as described before (6). Briefly, 30µg protein samples were resolved onto SDS-PAGE, with polyacrylamide (Sigma-Aldrich A3699) percentages suitable for the analyzed protein size. Proteins were transferred to a nitrocellulose membrane (Sartorius 11306). Membranes were then incubated with a primary antibody diluted in the same buffer, either at room temperature for one hour or overnight at 4°C. Upon incubation completion, membranes were washed three times with PBS containing 0.05% Tween 20 (PBST) (Sigma-Aldrich PPB005). A secondary antibody attached to peroxidase was diluted in a blocking buffer for one hour at room temperature. Detection of proteins specifically reacting with a primary antibody was carried out using a chemiluminescence-enhancing system (Merck-wblu0500) (23) in accordance with the manufacturer’s instructions. Quantification and comparative intensity of the relevant WB bands were determined using the Image-J software (http://image.net/).

### 2.6 Antibodies

The following antibodies were used in the current work: affinity purified polyclonal anti-N-terminal Fer (anti-N-Fer, directed toward amino acids 1–189, of Fer), and anti-Fer -SH2 antibodies that were generated in our lab (6, 7)., anti-ATP5B mitochondrial ATP synthase beta subunit monoclonal antibody (Santa Cruz Biotechnology), anti-phospho-AMPK (Thr172) (Cell Signaling CST-#4188), anti-AMPKα1 (Santa Cruz Biotechnology sc-398861), anti -phospho-Raptor (Ser792) (Cell Signaling CST-2083P), anti-Raptor (Cell Signaling CST-9862T), anti-phospho-mTOR (Ser2448), anti-mTOR, anti-phosphoTSC2 (Ser664), and anti-TSC2 (the mTOR Pathway Antibody Sampler Kit, Cell Signaling CST-9862T), anti-phospho-ERK1/2 (Thr202,Tyr204) (Sigma-Aldrich SAB4301578), anti-ERK1/2 (Abcam ab17942), anti-phospho-MEK1/2 (Ser218/222)- (Santa Cruz Biotechnology sc7995), anti-MEK1/2 (Santa Cruz Biotechnology sc-436), anti-NDUFA9 (Abcam-ab55521), anti-ATP5A (Abcam ab176569), anti-S6K (Abcam- ab186753), anti-phospho-S6K (Thr389) (Abcam ab32359), anti-actin (Santa Cruz Biotechnology sc-8432), and anti-α tubulin antibody (Santa Cruz Biotechnology sc-8035).

### 2.7 Determination of cellular ATP level

Cells were left untreated or treated with E260 for 48 h. Whole cell lysates were then prepared from 10^6^ viable cells using deproteinizing preparation kit (BioVision K808–200). ATP level was determined using the Abcam ATP-assay kit (ab 83355) according to the manufacturer instructions.

### 2.8 Determination of cellular aspartate level

Cells were left untreated or treated with E260 for 48 and 72 hr. Cell were then harvested and extracts were prepared from viable cells. Aspartate levels were determined using the Abcam Aspartate Assay Kit (ab102512) according to the manufacturer instructions.

### 2.9 Transmission electron microscopy

Post-treatment cells were incubated overnight in Karnovsky fixation solution (https://protocols.lo/view/karnovsky-39-s-fixative-ckiguubw), after which they were washed in 0.1 M cacodylate buffer and fixated in 1% OsO_4_ buffer for one hour. The preparations were then dehydrated using ascending alcohol concentrations (70–100%) and propylene oxide solution, and then fixed in Mix Agar. Thin (60mm) slices were cut and dyed in uranyl acetate and lead citrate to enhance the contrast, and were then examined under a FEI Tecnai transmission electron microscope (TEM).

### 2.10 Immunocytochemistry

Cells were fixated for 30 minutes in a 4% paraformaldehyde (PFA) (Sigma-Aldrich P6148) fixation solution prepared in PBS, and were then washed 3 times in PBS for 35 minutes at room temperature, to remove the PFA. The cells were then incubated with a blocking solution for 40 minutes, followed by 3 irrigations with PBST. Samples were incubated with a primary antibody diluted in blocking solution for one hr at room temperature and washed 3 times in PBST. From this stage onward all steps were carried out in darkness. The samples were incubated with a secondary antibody linked to a florescent marker diluted in antibody blocking solution for 40 minutes, and washed 3 times with PBST. Nuclei were stained for 10 minutes with the Hoechst DNA dye (2mg/ml solution), and were then rinsed 3 times in PBST. Samples where over-laid with an anti-fading cover, and then covered with a protective glass lid and kept at 4°C until being inspected by a confocal microscope (Olympus FV1000).

### 2.11 SiRNA transfection and knockdown of Fer

Transfections were carried out using the INTERFER in - siRNA transfection reagent (Sigma-Aldrich Inc.) according to the manufacturer’s instructions. Briefly, 10^5^ cells were seeded in 6 mm plates supplied with MEM containing 2 mM L-glutamine, and were subjected to 150 nM control siRNA, or siRNA directed selectively toward the *fer* mRNA (Sigma-Aldrich Inc.) (6, 7), suspended in 150 μM transfection reagent, for 72 hr. Cells were then harvested and lysates were subjected to WB analysis.

### 2.12 Bioinformatic analysis

The expression data were downloaded from UCSC Xena (https://xena.ucsc.edu/, PMID= 32444850). The analyzed data included gene expression data of PDAC tumor samples from the cancer genome atlas (TCGA) and gene expression data of healthy samples from the Genotype-Tissue Expression (GTEx) downloaded from UCSC Xena data hubs (PMID= 28398314). Specifically, the data used in the gene expression correlation analysis were obtained from the ‘UCSC Toil RNAseq Recompute’ data hub. Under this data hub, from “TCGA TARGET GTEx” cohort, the RSEM_norm_count file was downloaded from the “gene expression RNAseq” section, and the TCGA_GTEX_main_categories file was downloaded from the “phenotype” section. The samples for which ‘TCGA_GTEX_main_category’ was; “GTEX Pancreas” or “TCGA Pancreatic Adenocarcinoma” were selected.

### 2.13 Statistical analysis

Statistical analysis was performed using the paired Student’s t-tests, with a P<0.05 being considered significant. Results are depicted as mean ± standard deviation of the mean for n given samples.

## 3 Results

### 3.1 The E260-Fer inhibitor evokes death in PDAC cells

In order to examine and quantify the effect of E260 on PDAC cells, the effect of the compound on the viability of PANC-1, pancreatic cancer cells, was measured using MultiTox-Fluor Multiplex Cytotoxicity assay. As seen in **Supplementary Figure 1A**, subjection of the PANC-1 cells to ascending concentrations of E260 for 24 hr decreased the cell viability down to 45–50%. The effective concentration 50 (EC50) of E260 was found to be 0.58µM (**Supplementary Figure 1A**).

**Figure 1:**
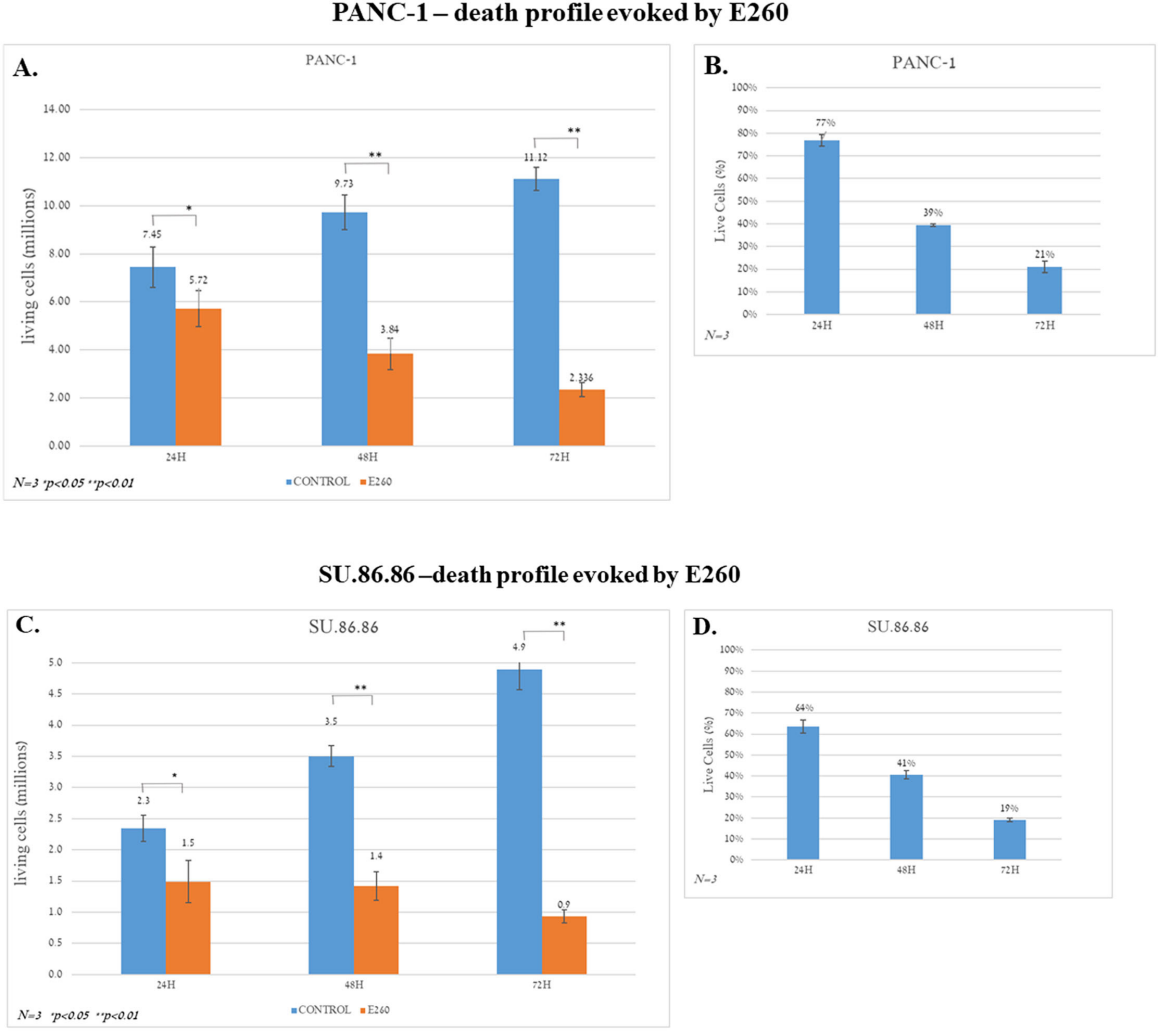
Death profiles evoked by E260 in PDAC cells. **(A)** PANC-1 cells were subjected to 5 µM E260 for 24, 48, and 72 hr. The number of live cells in each time point was determined using an automatic cell counter after the addition of Trypan blue to the samples. **(B)** The percentage of live cells is also shown. **(C, D)** Similar experiments and analyses were carried out with the SU.86.86 cells that were subjected to 2.5 µM, for 24, 48, and 72 hr. Data represent average values of three independent experiments that gave similar results. Standard deviations and *P* values are presented.

When the same analysis was conducted with cells exposed to E260 for 48 hr, a greater decline of cell viability, down to 30%, was already achieved at a relatively low concentration of E260 (0.5µM), and the measured EC50 was 0.35µM (**Supplementary Figure 1B**). Cells exposed to E260 for 72 hr showed almost complete cell death, and their viability already declined to 10% at the lowest concentration (0.5µM) and the EC50 decreased to 0.25µM, respectively (**Supplementary Figure 1C**). Similar, statistically significant profiles were obtained when the non-metastatic and metastatic, PANC-1 and SU.86.86 PDAC cells respectively, were treated with E260 for 24, 48, and 72 hr, and analyzed in a cell viability counting assay (**Figures 1A–D**). These results indicate that E260 requires time to induce cell death in pancreatic cancer cells, reaching death onset in 80% of the treated cells, when the cells are subjected to the E260-Fer inhibitor, for 72 hr.

**Figure 2:**
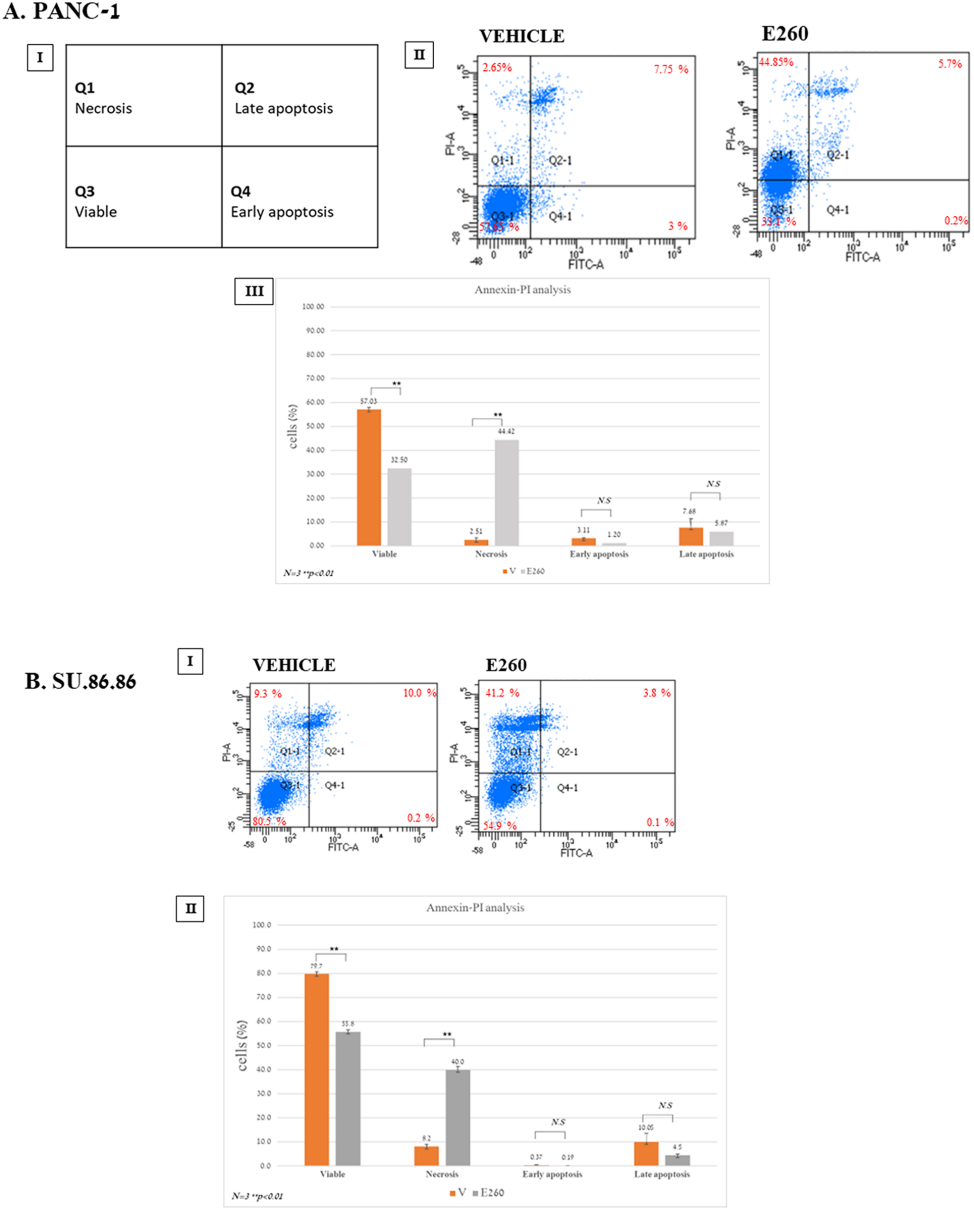
E260 evokes necrotic death in PDAC cells. **(A)** PANC-1 (I-III), and **(B)** SU.86.86 (I, II) cells were left untreated or subjected to 5 µM, or 2.5 µM E260, respectively, for 48 hr. Cells were stained with Annexin V-FITC and PI. Staining was quantified by FACS ARIAIII. All data were analyzed using FlowJo software. AIII and BII present respective average values of PANC-1 and SU.86.86 cell cohorts, obtained from three independent experiments that gave similar results. Standard deviations and *P* values are presented.

To characterize the death type evoked in the E260 treated PDAC cells, PANC-1 and SU.86.86. cells were incubated for 48 hr with 5 and 2.5 µM E260, respectively. These concentrations were selected based on the results obtained in the death levels analysis presented above (**Figure 1**, **Supplementary Figure 1**). Untreated and treated cells were then subjected to annexin-PI staining analysis. This revealed that in both PANC-1 and SU.86.86 cells subjected to Fer inhibition, the death evoked was rather necrotic and not apoptotic death (**Figures 2A, B**).

### 3.2 E260 affects mitochondrial morphology in PDAC cells

As Fer was shown to reside in the mitochondria of colorectal cancer cells (6, 7) we turned to verify its presence in the mitochondria of PDAC cells. Cells were subjected to immuno-cytochemical analysis using the specific anti-N-Fer antibodies directed toward Fer (6, 7), combined with an antibody directed toward the ATP5B protein that resides in the mitochondrion (24), and was therefore serving as a marker for this organelle. Fixed and immuno-stained cells were then inspected under confocal fluorescence microscopy. This demonstrated the presence of Fer in cytoplasmic sub-cellular compartments (**Supplementary Figures 3I, II**). Co-staining experiments specifically showed the localization of Fer in the mitochondria of PDAC cells (Yellow colored areas in the red and green merged panels (**Supplementary Figures 3III, VI**), indicating the localization of Fer to the mitochondria).

In order to confirm the necrotic death type evoked by E260 in PDAC cells, and to examine the effect of Fer-targeting on the mitochondria of the treated cells, untreated and treated cells were examined under transmission electron microscopy (TEM). The cells were treated with the E260 vehicle (6), or the E260 itself at 2µM, for 24, 28 and 72 hr. The cells were then fixed and prepared for examination under TEM.

**Figure 3:**
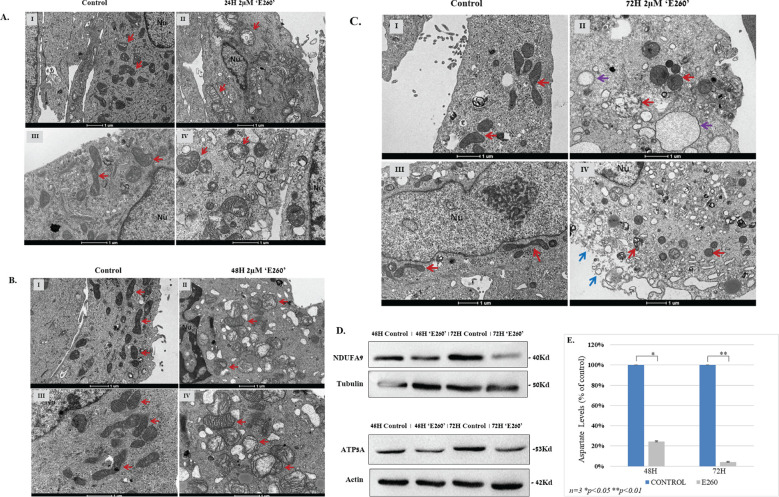
**(A)** TEM morphology images of PANC-1 cells exposed to E260 for 24 hr. Treated and fixed cells were inspected under TEM after vehicle treatment alone (I, III), or 2µM E260 for 24 hr (II, IV). Red arrows indicate mitochondria. Nu - cell nucleus. Magnification bars are given in the bottom of each image. **(B)** TEM morphology images of PANC-1 cells after 48 hr exposure to E260. PANC-1 inspected under TEM after vehicle treatment alone (I, III), or E260 treatment for 48 hr (II, IV). Red arrows indicate mitochondria. Nu - cell nucleus. Magnification bars are given in the bottom of each image. **(C)** TEM morphology images of PANC-1 cells after 72 hr exposure to E260. PANC-1 cell inspected under TEM after vehicle treatment alone (I, III), or E260 treatment for 72 hr (II, IV). Red arrows indicate mitochondria. Purple arrows denote autophagosomes, and blue arrows indicate disruption of the cells plasma membrane. Nu - cell nucleus. Magnification bars are given in the bottom of each image. **(D)** Cells were left untreated or subjected to E260 for 48 and 72 hr. Whole cell lysates were resolved in SDS-PAGE and reacted with: anti- NDUFA, and anti-ATP5A, in a WB analysis. **(E)** Lysates prepared from the cells in **(D)** were also taken for aspartate levels determination. Each panel of the above represent one out of three independent experiments that gave similar results is presented.

This analysis revealed that cells treated with E260 have deformed (inflated) mitochondria (**Figure 3**), thus indicating morphological deformation of these organelles in all treatment stages of the cells. Importantly, these defects were not seen in vehicle treated cells (**Figures 3A–C**). Morphological mitochondrial deformation was profoundly manifested when cells where exposed to the E260 compound for 48 hr. Mitochondria were undergoing swelling, a state which was not observed in cells treated with the drug solute alone (**Figure 3B**). After 72 hr treatment with E260, full mitochondrial damage was seen, as they look inflated and out of shape. In this period of time, the appearance of autophagosomes can be tracked as well (**Figure 3C**). Notably, disruption of cells’ outer plasma membrane is also seen after 72 hr (**Figure 3C**, blue arrows), corroborating the onset of necrotic, rather than apoptotic, death (25) in the treated cells. Accordingly, while outer cellular, plasma membrane is defected, no inner nuclear change is observed in the affected cells (**Figure 3C**). This also envisages the onset of necrotic, rather than apoptotic, death, which would involve chromatin condensation and nuclear fragmentation in the affected cells. To biochemically document the effect of E260 on the mitochondria of PDAC cells, we compared the levels of two mitochondrial markers, NDUFA9 and ATP5A, in untreated and E260 treated cells. These markers are constituents of the mitochondrial ETC- complex I (NDUFA9), and complex V (ATP5A) [8], and it can be clearly seen that their level is decreased in E260 treated cells (**Figure 3D** and **Supplementary Figure 3D**). Mitochondrial deformation is expected to jeopardize the mitochondrial respiratory and metabolic activities, thereby leading to deficiency in aspartate which is a key metabolic product of mitochondrial respiration, and an essential metabolite for cell viability and proliferation (26, 27). Indeed, subjection to E260 led to a significant decrease in aspartate level in the treated PDAC cells (**Figure 3E**), an effect that by itself can evoke cell death (26, 27).

### 3.3 The E260-Fer inhibitor affects key regulatory cascades that govern the mTORC1 metabolic hub in PDAC cells

**Figure 4:**
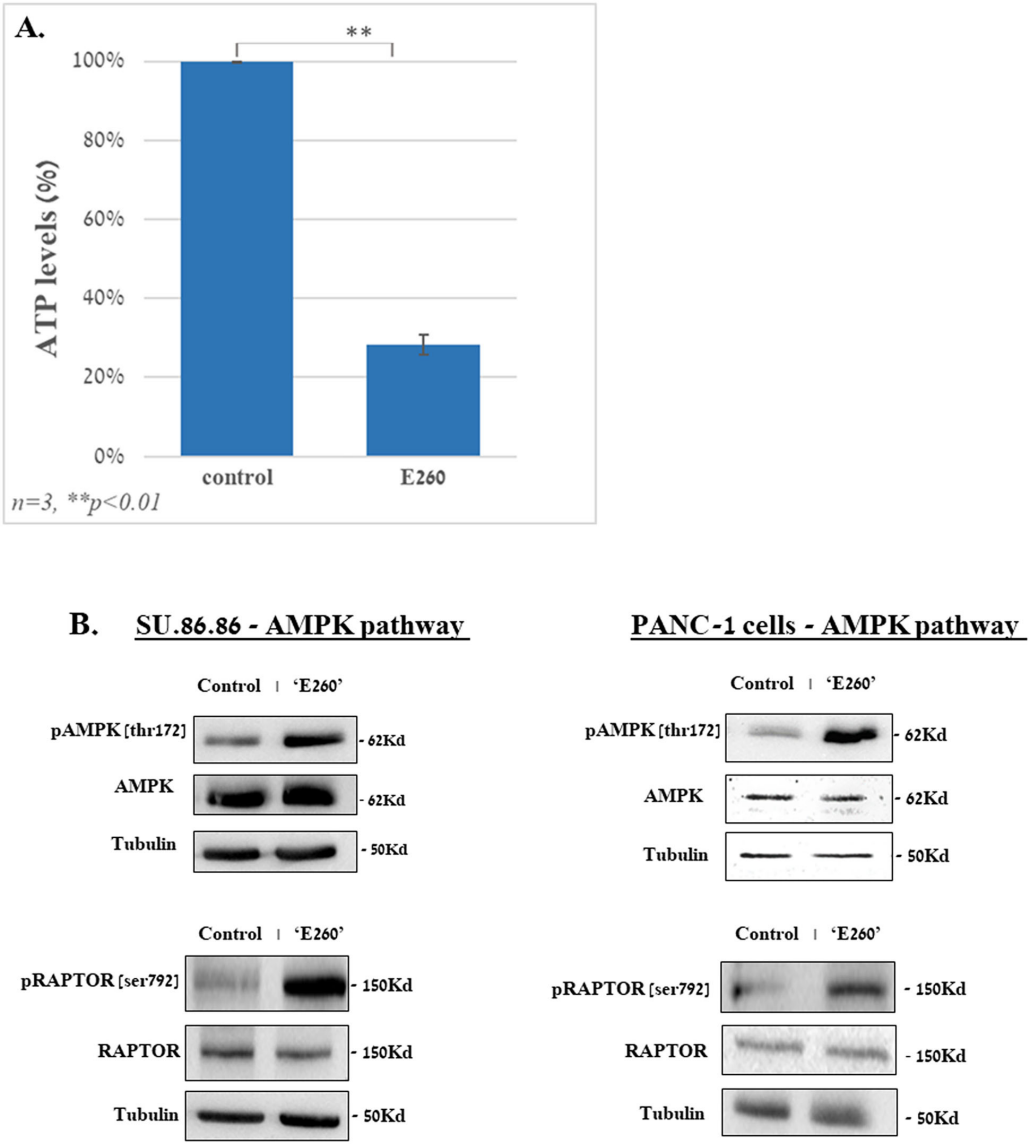
E260 leads to ATP depletion and activation of AMPK in PDAC cells. SU.86.86 and PANC-1 cells were left untreated or subjected to 2.5 µM E260 for 48 hr, in MEM supplied with 2mM L-glutamine. Cells were then harvested, and their lysates were, **(A)** subjected to ATP analysis, or **(B)** resolved in SDS-PAGE and reacted with: anti-phospho-AMPK (Thr. 172), anti-AMPK α1, anti-phospho-RAPTOR (Ser 792), anti-Raptor, and anti-tubulin, antibodies, in a WB analysis. One out of three independent experiments that gave similar results is presented.

**Figure 5:**
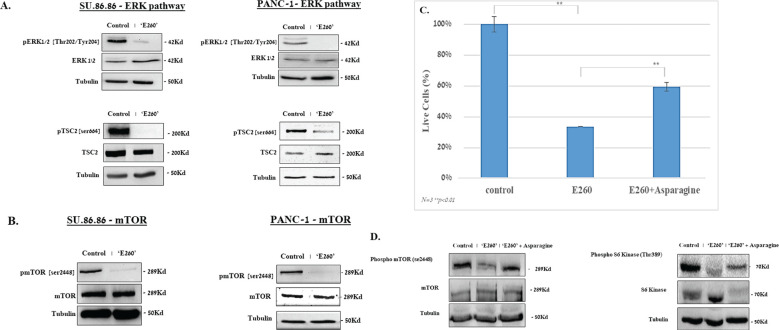
E260 inhibits ERK1/2 signaling cascade and mTORC1 activation in PDAC cells. SU.86.86 and PANC-1 cells were left untreated or subjected to 2.5 µM E260 for 48 hr, in MEM supplied with 2mM L-glutamine. Cells were then harvested, and their lysates were resolved in SDS-PAGE and subjected to **(A)** anti-pospho-ERK1/2 (Thr.202/Tyr204), anti-ERK1/2, anti-phspho-TSC2 (Ser 664), anti-TSC, and anti-Tubulin. **(B)** anti-phospho-mTOR (Ser 2448), anti-mTOR, and anti-Tubulin, in a WB analysis. **(C)** Asparagine alleviates the death level evoked by E260 in PDAC cells. SU.86.86 cells were left untreated or treated with 2.5 µM E260, in the absence or presence of 4 mM asparagine, for 48 hr. The percentage of viable cells in each sample was determined using an automatic cell counter after the addition of Trypan blue to the sample. Data represent average values of three independent experiments that gave similar results. Standard deviations and *P* values are presented. **(D)** Lysates from each sample were resolved in SDS-PAGE and reacted with: anti-phospho-mTOR (Ser 2448), anti-mTOR (left panel), anti- phosphor-S6K (Thr389), anti-S6K (right panel), and anti-Tubulin, in a WB analysis. In each panel, one out of three independent experiments that gave similar results is presented.

The profound effect of E260 on the mitochondria integrity (**Figure 3**) and function, leads to ATP depletion in the treated cancer cells (**Figure 4A**) (6). This is expected to activate the metabolic AMP/ADP/ATP ratio sensor AMP-activated protein kinase (AMPK), which senses the distorted balance between AMP/ADP and ATP (28). Treatment of PANC-1 and SU.86.86 cells with E260 led indeed to the activating phosphorylation of AMPK on threonine^172^ (29) (**Figure 4B**, **Supplementary Figure 4B**). Activation of AMPK enabled it to phosphorylate the mTORC1 regulatory subunit, RAPTOR, on serine^792^ (30) (**Figure 4B**, **Supplementary Figure 4B**). Phosphorylation of RAPTOR on this moiety leads to its dissociation from mTORC1 and to the consequent inactivation of mTOR (31, 32) (**Figure 5B**, **Supplementary Figure 5B**), which is the catalytic core of the mTORC1-key, regulatory metabolic hub (33, 34).

Additional regulators of the mTORC1 activity are the ERK1/2 kinases (35). We have shown before that Fer associates with ERK1/2 and sustains their activation state under abnormal cellular growth conditions, independently of the Fer tyrosine kinase activity (36). In relevance to this observation, E260 was found to affect both the kinase dependent and independent activities of Fer, by distorting the tertiary structure of the enzyme’s kinase domain (6). We therefore assumed that E260 could interfere with the effect of Fer on ERK1/2, and we turned to examine whether subjection of PDAC cells to E260 could affect the activation state of ERK1/2 in these treated cells. When either PANC-1 or SU.86.86 cells were treated with E260, this indeed led to a profound deactivation of ERK1/2, in both the non-metastatic and metastatic PDAC cells (**Figure 5A**, **Supplementary Figure 5AI**). Notably, E260 only mildly affected the phosphorylation level of the ERK1/2 upstream activators MEK1/2 (37) (**Supplementary Figure 5AII**), thus supporting the notion that E260 affects primarily ERK1/2. ERK1/2 can phosphorylate and neutralize the mTORC1 suppressor TSC2 (33, 38), thereby leading to the sustention of the mTORC1 activation state. Accordingly, the deactivation of ERK1/2 by E260 resulted in the dephosphorylation of the mTORC1 suppressor TSC2 on serine^664^ (**Figure 5A**, **Supplementary Figure 5AI**), which is a direct substrate of these serine/threonine kinases (38). It should be noted that the activation state of another putative regulator of TSC2, namely the AKT kinase (39), was not affected by E260 (**Supplementary Figure 5AIII**), corroborating the notion that the effect of E260 on TSC2 is mediated primarily through deactivation of ERK1/2. Since the dephosphorylation of TSC2 on serine^664^ resumes its mTOR suppressive activity (34), this also led to the deactivation of mTORC1, as was manifested by the dephosphorylation of mTOR on serine^2448^ (**Figure 5B**, **Supplementary Figure 5B**), a phosphorylation site that serves as an activation state marker, of the mTOR complex (40).

The mitochondrial damage caused by E260, and the parallel downregulation of mTORC1 that plays an important role in mitochondrial biogenesis and onco-metabolism (21), can converge and lead to the onset of death in the treated PDAC cells. To examine the causal role of mTORC1 deactivation in the death evoked by E260 in PDAC cells, we subjected the cells to E260 in the absence or presence of externally supplied asparagine. The asparagine amino acid (aa) was shown to restore mTORC1 activity in the context of mitochondrial damage and electron transport chain inhibition (22). It is thus expected to alleviate the death level evoked in the E260 treated PDAC cells. Addition of asparagine did increase the percentage of viable cells that survive the E260 treatment (**Figure 5C**). In compliance with this observation, asparagine also restored the basic activation state of mTORC1 (**Figure 5D**, **Supplementary Figure 5D**), and its bona fide substrate and downstream effector-S6 Kinase (S6K) (19) (**Figure 5D**, **Supplementary Figure 5D**), under E260 treatment conditions. Hence, deactivation of mTORC1 contributes to the onset of death in E260 treated PDAC cells.

### 3.4 Knockdown of Fer leads to the activation of AMPK and to the deactivation of ERK1/2 and mTORC1 in PDAC cells

**Figure 6:**
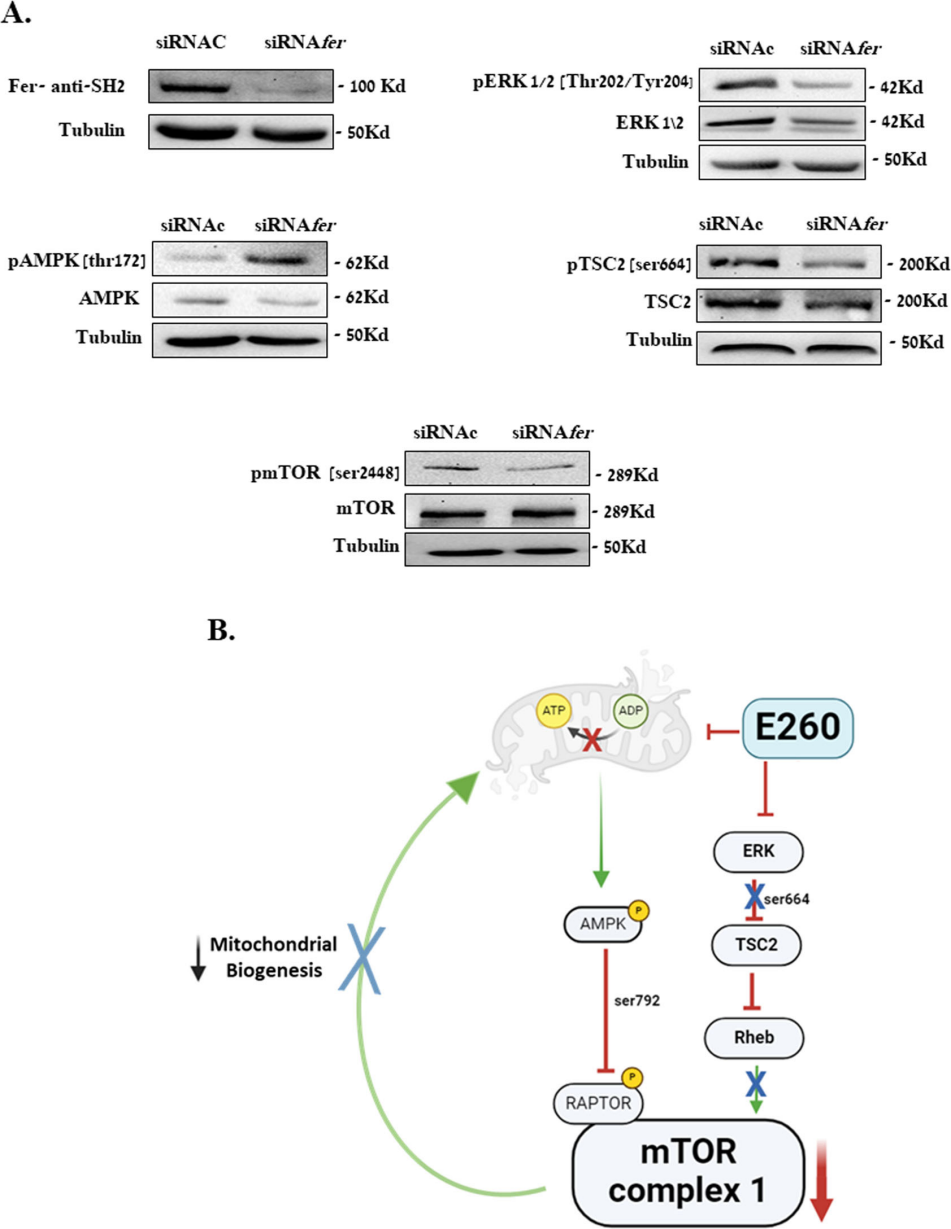
Knockdown of Fer activates AMPK and downregulates ERK1/2 in PDAC cells. **(A)** SU.86.86. cells were transfected with control siRNA (siRNAc), or *fer*-targeting siRNA (siRNA*fer*) and incubated in MEM supplied with 2 mM L-glutamine, for 72 hr. Cells were then harvested, and their lysates were resolved in SDS-PAGE and subjected to: anti-Fer (SH2), anti-pospho-ERK1/2 (Thr.202/Tyr204), anti-ERK1/2, anti-phspho-TSC2 (Ser 664), anti-TSC, anti-phospoh-AMPK (Thr172), anti-AMPK, anti-phospho-mTOR (Ser 2448), anti-mTOR, and anti-Tubulin, in a WB analysis. One out of three independent experiments that gave similar results is presented. **(B)** Summarizing scheme depicting the two regulatory pathways affected by E260 and converging to the downregulation of mTORC1 and mitochondrial function, in PDAC cells.

To establish the functional link between the effects of E260 on key metabolic hubs and the regulatory roles of Fer in PDAC cells, we carried out knockdown experiments, in which the level of Fer was decreased in SU.86.86 cells using specific and selective siRNA directed toward the *fer* mRNA (6, 7). As was seen for E260, this led to the activation of AMPK and to the deactivation of ERK1/2 (**Figure 6A**, **Supplementary Figure 6A**). Importantly, the knockdown effect of Fer on these two key regulators culminated in the reduced activation level of the mTOR complex in the Fer depleted cells (**Figure 6A**, **Supplementary Figure 6A**). Similar results were obtained when the level of Fer was knocked-down in PANC-1 cells (**Supplementary Figure 6B**). Thus, the effects of E260 on AMPK, ERK1/2 and mTORC1 can be directly linked to the modulatory, and sustaining roles of Fer in the signaling cascades in which these regulators are engaged (**Figure 6B**).

### 3.5 The expression level of Fer shows positive correlation with the expression level of mTOR and its downstream effector- LARP1- in PDAC tumors

**Figure 7:**
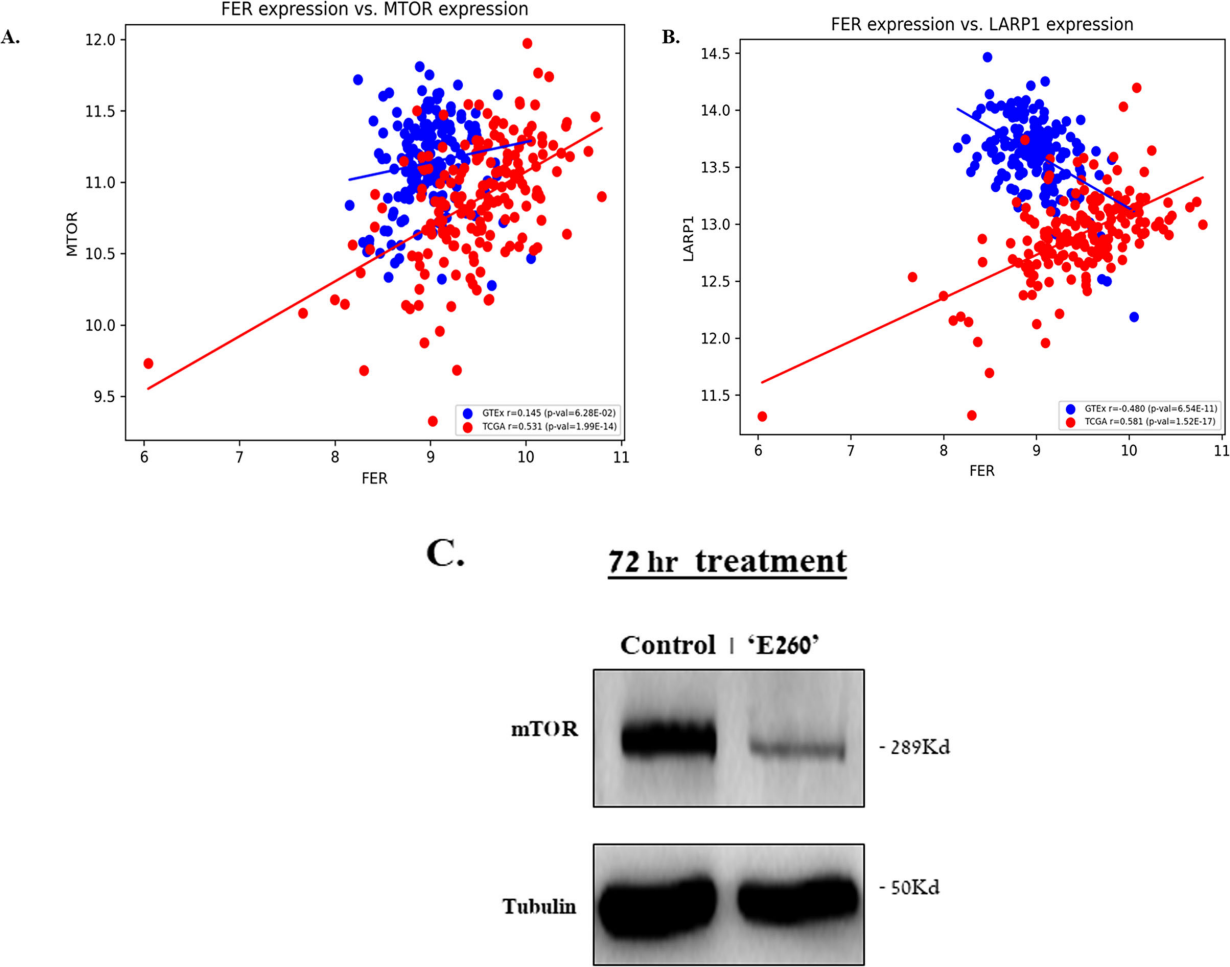
The expression level of Fer shows positive correlation with the expression level of mTOR and LARP1- in PDAC tumors. Scatter plot of Fer vs mTOR expression **(A)**, and Fer vs LARP1 expression levels **(B)**, in PDAC tumors taken from TCGA, and in normal human pancreatic tissue taken from GTEX. The Pearson correlation r and the p values are given. **(C)** SU.86.86 cells were left untreated or subjected to 2.5 µM E260 for 72 hr. Lysates resolved in SDS-PAGE were subjected to anti-mTOR, and anti-Tubulin, in a WB analysis. One out of three independent experiments that gave similar results is presented.

The suppressive effect of E260 on the activation state of the mTORC1 substrate and downstream effector -S6K (**Figure 5D**, **Supplementary Figure 5D**), reinforces the notion that downstream effectors of mTORC1 are down-regulated upon the targeting of Fer. Another direct substrate and downstream effector of mTORC1 is the La-related protein 1 (LARP1) which selectively stabilizes mRNAs encoding proteins essential for protein synthesis (41), including the mTOR mRNA itself (42). We therefore turned to examine whether the mRNA level of Fer could be correlated with the mRNA level of mTOR in pancreatic cancer tumors. Pearson correlation was calculated for the expression levels of the gene pairs – *FER* and *mTOR* and *FER* and *LARP1* in both tumor and healthy tissues (**Figures 7A, B**). While in PDAC tumor tissue (TCGA), the expression levels of Fer displays moderate positive correlation with a significant statistical significance with the expression levels of mTOR (r= 0.531, p-val=1.99E-14), in healthy tissue (GTEx) it does not show significant correlation (r= 0.145, p-val= 6.28E-02) (**Figure 7A**). This indicates the greater strength of the Fer vs. mTOR relationship in PDAC tumors. The correlation between Fer and mTOR expression levels in PDAC tumors, envisaged also a link between Fer and LARP1 in these malignant cells. To examine this assumption, Pearson correlation was also calculated for the expression levels of the genes *FER* vs *LARP1*. While in tumor tissue (TCGA), the expression levels of Fer displays moderate but statistically significant positive correlation with the expression levels of LARP1 (r=0.581, p-val=1.52E-17), in healthy tissue (GTEx) it displays a moderate negative correlation (r= –0.480, p-val= 6.54E-11) (**Figure 7B**). These findings suggest that downregulation of Fer could also affect the expression level of mTOR in PDAC cells. In compliance with this notion, an extended subjection (72 hr) to E260, did lead to a significant decrease in the mTOR level in the treated PDAC cells (**Figure 7C**, **Supplementary Figure 7C**).

## 4 Discussion

PDAC is one of the deadliest cancers with a high percentage of morbidity. The deciphering and identification of novel targets and tools for intervening with its adverse malignant growth are therefore of profound importance. Oncogenic KRAS is a main driver of PDAC development, progression, and metastasis. It leads to persistent activation of downstream signaling pathways of which the Raf/MEK/ERK (37, 43) and mTORC1 (14, 15) cascades are major constituents. Yet, selective targeting of each of these cascades did not lead to satisfactory clinical outcomes in PDAC patients (44).

In the current work we show that the intracellular tyrosine kinase-Fer sustains key regulatory processes and signaling pathways, some of which serve as downstream effectors of oncogenic KRAS in PDAC tumors. Accordingly, subjecting PDAC cells to the Fer targeting inhibitor E260 simultaneously affects two major subcellular target sites, culminating in the death of the challenged cells. One target site is the mitochondria, and the other one is the ERK1/2 growth promoting signaling cascade. It is well appreciated that the metabolic and energy generation systems are reprogrammed and modified in cancer cells. This enables malignant cells to cope with unique needs and challenges faced by these abnormal cells during their growth and spreading.

Although the relevance of aerobic glycolysis to cancer cell growth and metabolism (the “Warburg Effect”), has been well documented, recent studies have also established the importance of mitochondrial, energy and metabolic production processes for malignant transformation and metastasis (45). However, the relative importance and dependence of malignant cells on the mitochondrial Oxphos. varies among different types of cancers. Notably, PDAC tumors were shown to depend on an ongoing and active mitochondrial oxidative phosphorylation (8). Thus, targeting the mitochondria of PDAC cells bears with it a novel tool for the treatment of this deadly disease. Indeed, in the current work we show that targeting Fer, which is a key constituent of the reprogrammed mitochondria of malignant cells, leads to profound effects in the treated PDAC cells. Not only is the function of the mitochondria affected, they also undergo swelling and deformation. Such an effect decreases the cellular level of ATP, and leads to the activation of the AMP/ADP/ATP ratio sensor AMPK (6). Accordingly, we show here that E260 leads to the activation of AMPK in PDAC cells. This leads to the phosphorylation of the AMPK substrates – RAPTOR, and to the inactivation of mTORC1 due to the dissociation of the phosphorylated RAPTOR from the core, metabolic hub complex (31, 32). Notably, an additional, parallel pathway that leads to the deactivation of mTORC1 is being affected by E260, through the inactivation of ERK1/2. This inhibitory effect of E260 on ERK1/2 coincides with the presence of Fer in the extra-mitochondrial cytoplasmic compartment of PDAC cells (**Supplementary Figures 3I, II**), and with its association with ERK1/2 for sustaining the activation state of these kinases under abnormal growth conditions (36). Since E260 distorts the tertiary structure of the Fer kinase-domain (6), it could dissociate Fer from ERK1/2, thereby subjecting these signaling regulators to cellular dephosphorylating activities (46). The dual effects of E260 on the mitochondria and ERK1/2 culminate in the deactivation of mTORC1 and its downstream effector S6K (19), an outcome that contributes to the death evoked by E260 in the therapy refractory, metastatic PDAC cells. mTORC1 serves as a metabolic hub that governs and stimulates numerous anabolic processes, like protein, nucleotide, and lipid syntheses (47), which are vital for cell-growth and cancer progression. In addition, mTORC1 regulates mitochondrial integrity and function by directing mitochondrial biogenesis, glutaminolysis, and the generation of mitochondrial oncometabolites (21). Hence, the direct targeting of mitochondria by E260 on one hand, combined with the E260 driven downregulation of mTORC1on the other hand, expose the treated PDAC cells to impaired key metabolic processes like severe aspartate deficiency (26, 27) (**Figure 3E**), and to a consequent onset of cell-death. The converging effects of E260 on mitochondria, and on the key metabolic hub mTORC1, has an efficacious therapeutic outcome that is expected to withstand potential cellular cricumventing pathways, and to defy resistance acquirement by the E260 treated PDAC cells. The functional link between Fer and the sustained activation of mTORC1 in PDAC cells, is manifested by the positive correlation between Fer and the mTOR expression level, in PDAC tumors. Furthermore, we show here for the first time that the expression level of Fer is positively correlated, in PDAC tumors but not in normal pancreatic tissues, to the elevated expression of the downstream effector and regulator of mTORC1 -LARP1. Since LARP1 was shown to act as an onco-protein that propells malignant proliferation, invasion, and metastasis (42), our findings open new avenues for deciphering the mTORC1 mediated role of Fer in potentiating the aggressive nature of PDAC tumors. On the top of the said above, it should be taken into account that Fer might also support the malignant phenotype of PDAC tumors through the sustension of growth, and survival promoting regulatory pathways, which are governed and propelled by ERK1/2, independently of mTORC1 (48, 49). In either prospect, the fact that the Fer targeting E260 compound efficaciously and selectively kills PDAC cells, without affecting normal cells is of note (6).

Overall, we show in this work that a Fer inhibitor can simultaneously target the mitochondria and a key metabolic hub like mTORC1, in PDAC cells. Notably, E260 affects also the ERK1/2 kinases and leads to their deactivation in PDAC cells. Since these kinases are key downstream effectors of oncogenic KRAS which by itself is a main driver of PDAC development, progression, and metastasis, the targeting of ERK1/2 turns E260 into a unique and potent anti-cancer compound that in parralel, targets several regulatory arms which are essential for PDAC tumors development and progression (37, 43). This may open the way for applying E260 as an efficatious mono-therapy anti-PDAC agent, but may also raise concerns about its safety. Several anti-PDAC ERKs inhibitors failed in the clinic due to poor tolerability (50). However, unlike these inhibitors, E260 does not seem to inhibit ERK1/2 directly, but it rather targets the kinase-activity independent sustaining effect of Fer, on the ERK1/2 activation state. This effect of Fer was shown by us to occur primarily under abnormal growth conditions (36), an observation that coincides with the safety and lack of cytotoxic effects of E260 toward normal- primary human fibroblasts, epithelial cells, and hematopoietic stem cells (6). Collectively, it can be appreciated that simultaneous targeting of the mitochondria integrity and function on one hand, and the ERK1/2 and mTORC1 regulatory axes on the other hand, may open new direction for efficacious treatment of PDAC tumors. The efficacy, selectivity and safety of this approach should be confirmed and reinforced *in-vivo* in PDAC-PDX animal models, and in controlled pre-clinical animal toxicity studies.

## Data Availability

The original contributions presented in the study are included in the article/**Supplementary Material**. Further inquiries can be directed to the corresponding author.
