# Supplementary material for: Fer governs mTORC1 regulating pathways and sustains viability of pancreatic ductal adenocarcinoma cells
Source: Front Oncol. 2024 Aug 14;14:1427029. doi: 10.3389/fonc.2024.1427029 (PMC11349523; doi:10.3389/fonc.2024.1427029)

Supplementary

<u>Figure S1 .</u>

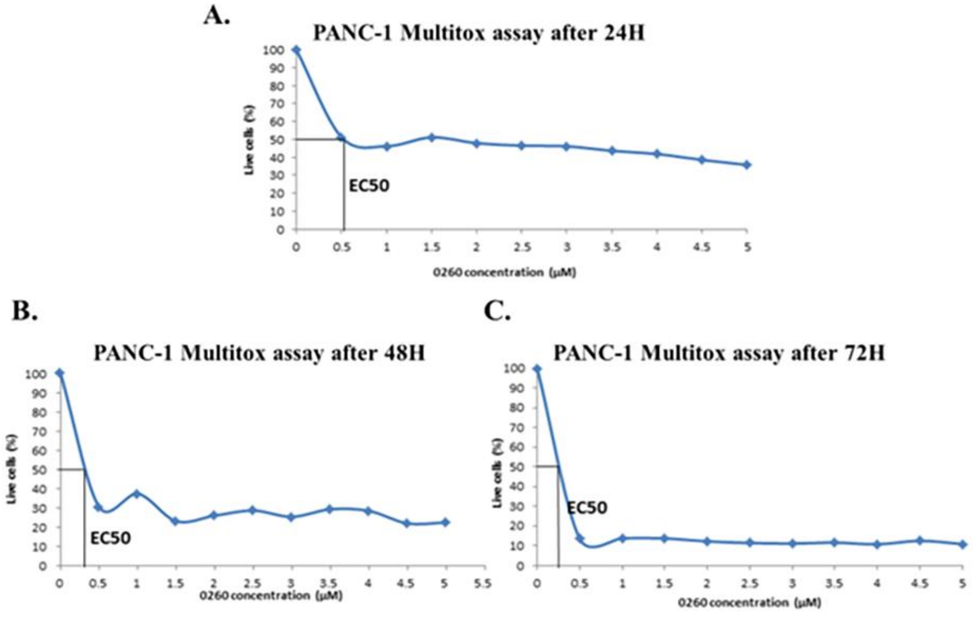

Assessing susceptibility of PDAC cells to ascending concentrations of E260 . Examination of PANC-1 cell viability after 24, 48 and 72 hr (**A**, **B.** and **C.**, respectively) following E260 treatment, in increasing concentrations. The percentage of viable cells was determined using the Multitox cell-viability assay, and calculated compared to the non-treated control. These calculations represent an average of three independent experiments that gave similar results.

Figure S3 .

Fer resides in the mitochondria of PCAD cells

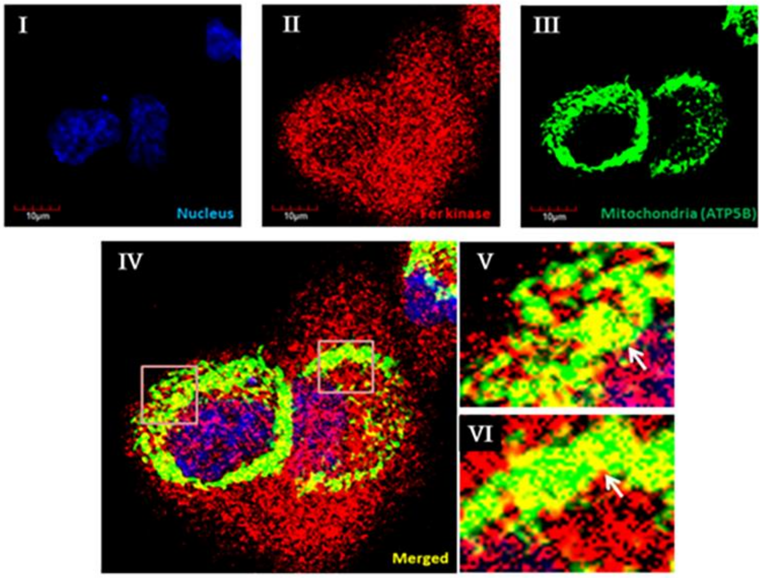

Figure S3. Fer resides in the mitochondria of PDAC cells. I.-III. PANC-1 cells exposed to immuno-cytochemical analysis of the mitochondrial marker ATP5B (green) and Fer (red). Cell nuclei were stained with the DNA dye-Hoechst (blue). **IV**. merged picture displaying the overlap between the location of Fer and the mitochondrial marker ATP5B (yellow). (**V.-VI.**) areas marked in squares in **IV**. under greater magnification.

Arrows point to co-localizations (yellow).

Figure S3D .

quantifications (su.86.86)

NDUFA9 normalized toTubulin

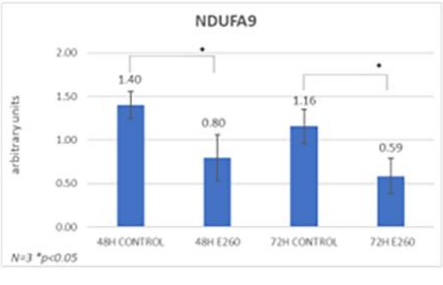

ATP5A normalized to Actin

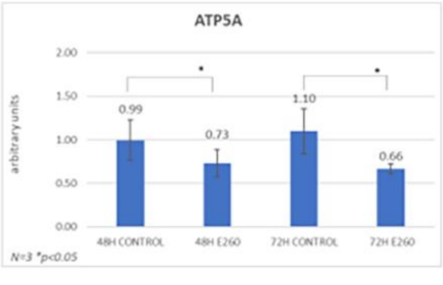

Figure S4B .

quantifications (su.86.86)

pAMPK normalized to AMPK and Tubulin

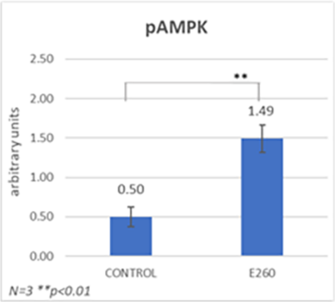

pRAPTOR normalized to RAPTOR and Tubulin

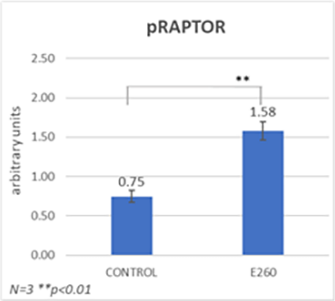

quantifications (PANC-1)

pAMPK normalized to AMPK and Tubulin

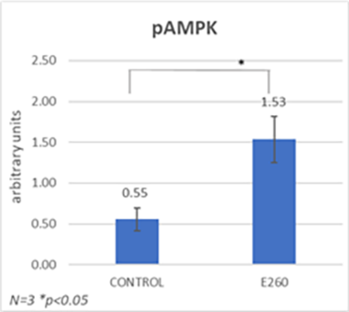

pRAPTOR normalized to RAPTOR and Tubulin

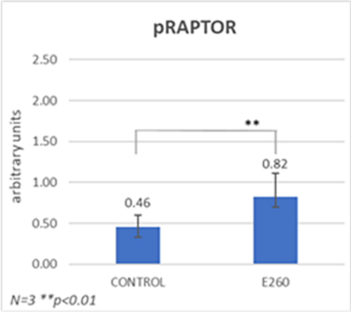

Figure S5AI .

quantifications (su.86.86)

pERK normalized to ERK and Tubulin

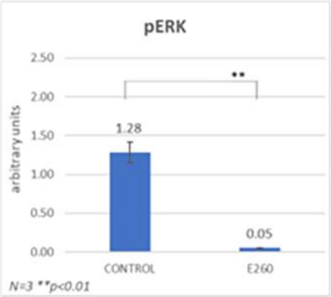

pTSC2 normalized to TSC2 and Tubulin

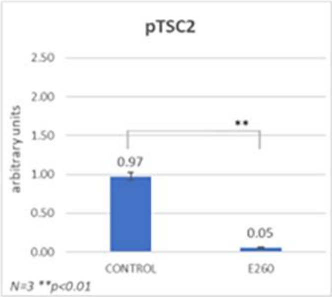

quantifications (PANC-1)

pERK normalized to ERK and Tubulin

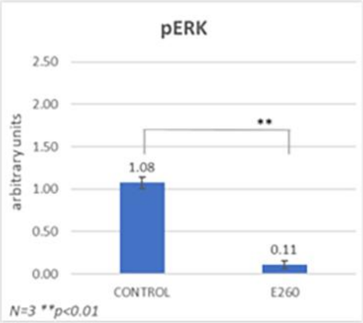

pTSC2 normalized to TSC2 and Tubulin

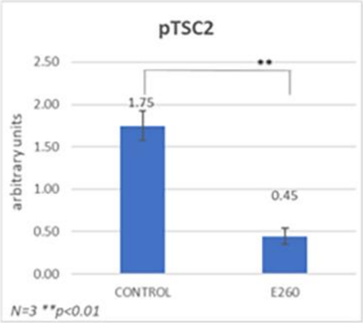

Figure S5AII .

Effect of E260 on MEK1/2

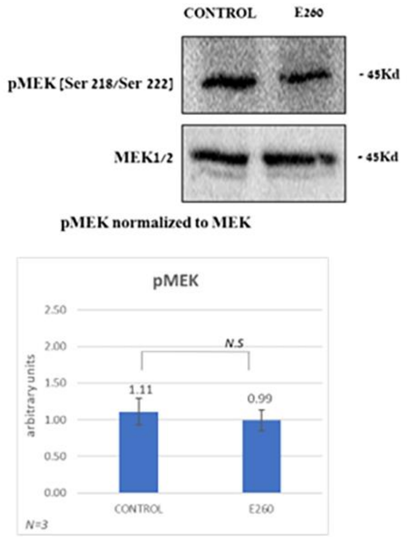

Figure S5AII . Effect of E260 on the upstream ERK1/2 activators-MEK1/2. SU.86.86 cells were subjected to 2.5 µM E260 for 48 hr, in MEM supplied with 2mM L-glutamine. Cells were then harvested, and their lysates were resolved in SDS-PAGE and subjected to anti-pospho-MEK1/2 (Ser218/222, upper panel), and anti-MEK1/2 (lower panel). One out of three independent experiments that gave similar results is presented.

Figure S5AIII .

Effect of E260 on AKT

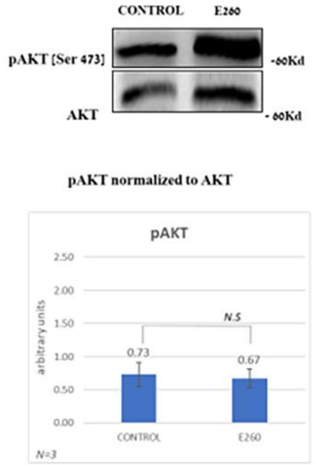

Figure S5AIII . E260 does not affect the AKT kinase activation state. SU.86.86 cells were left untreated or subjected to 2.5 µM E260 for 48 hr, in MEM supplied with 2mM L-glutamine. Cells were then harvested, and their lysates were resolved in SDS-PAGE and subjected to anti-pospho-AKT (Ser473, upper panel), and anti-AKT (lower panel) in a WB analysis. One out of three independent experiments that gave similar results is presented.

Figure S5B .

quantifications (su.86.86)

pmTOR normalized to mTOR and Tubulin

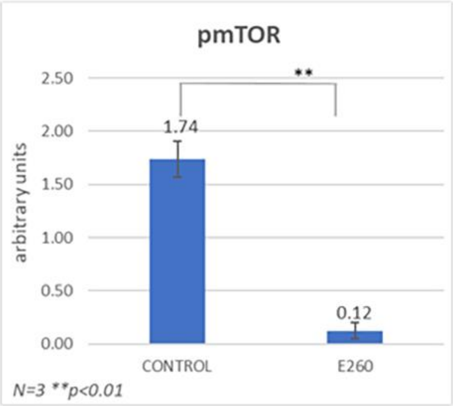

quantifications (PANC-1)

pmTOR normalized to mTOR and Tubulin

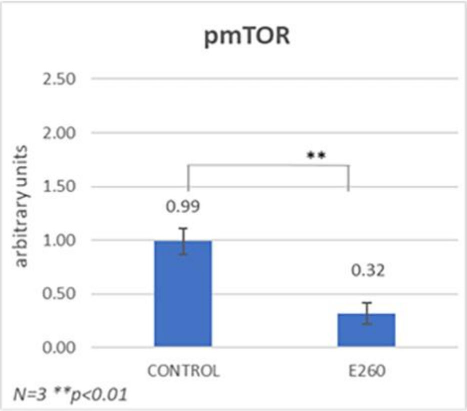

Figure S5D .

quantifications (su.86.86)

pmTOR normalized to mTOR and Tubulin

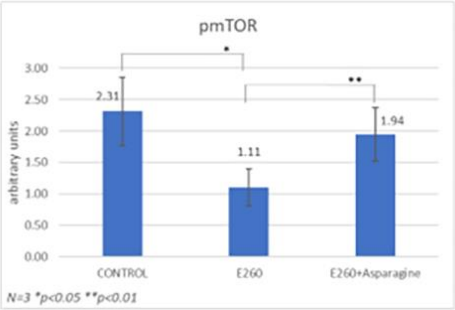

pS6K normalized to S6K and Tubulin

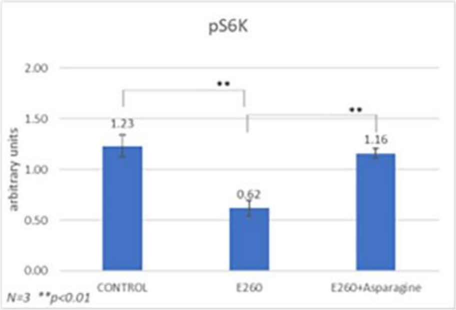

Figure S6A .

quantifications (su.86.86 - siRNA *fer*)

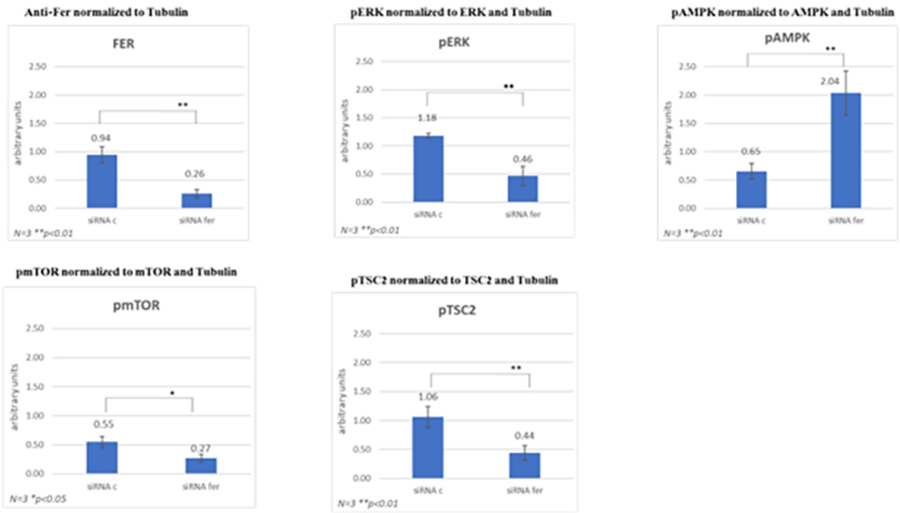

Figure S6B .

PANC-1, Si-RNA *fer*

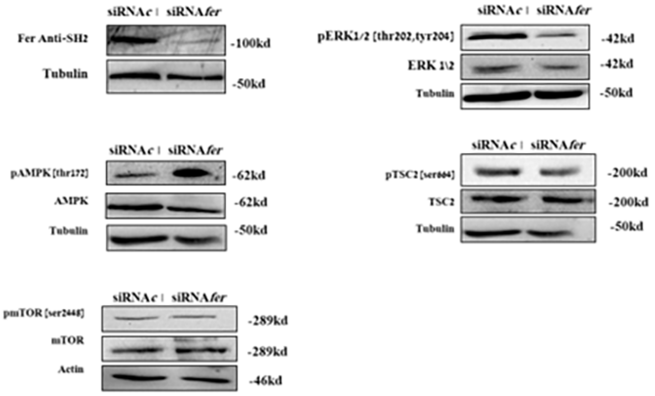

Figure S6B. Knockdown of Fer activates AMPK and downregulates ERK1/2 in PANC-1 PDAC cells. PANC-1 cells were transfected with control siRNA (siRNAc), or *fer*-targeting siRNA (siRNA*fer*) and incubated in MEM supplied with 2 mM L-glutamine, for 72 hr. Cells were then harvested, and their lysates were resolved in SDS-PAGE and subjected to: anti-pospho-ERK1/2 (Thr.202/Tyr204), anti-ERK1/2, anti-phspho-TSC2 (Ser 664), anti-TSC, anti-phospoh-AMPK (Thr172), anti-AMPK, anti-phospho-mTOR (Ser 2448), anti-mTOR, and anti-Tubulin, in a WB analysis. One out of three independent experiments that gave similar results is presented.

Figure S7C .

Quantifications (su.86.86)

mTOR normalized to Tubulin

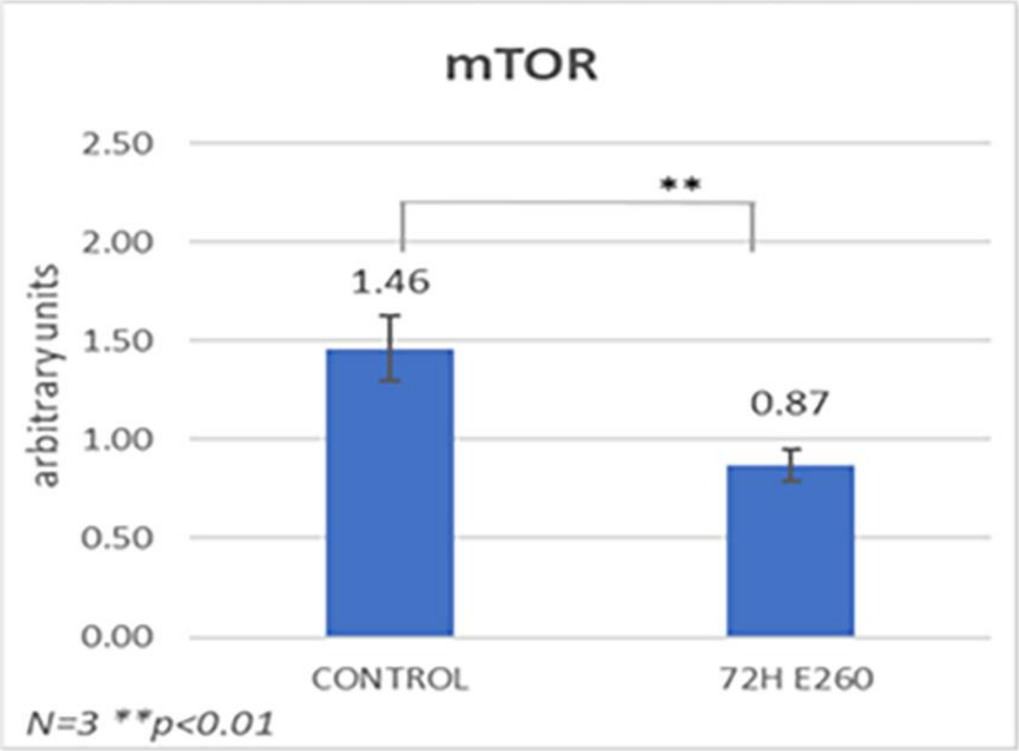

Supplement: Supplementary file 1 [file DataSheet1.pdf]
